# Epigenetic modifications of HIV proviral LTRs: potential targets for cure

**DOI:** 10.1186/1742-4690-9-S1-O4

**Published:** 2012-05-25

**Authors:** W Doerfler, S Weber, K Kemal, B Weiser, K Korn, K Anastos, H Burger

**Affiliations:** 1 Erlangen University, Institute for Virology, Erlangen, Germany; 2 Wadsworth Center, New York State Department of Health, Albany, NY USA; 3 Albert Einstein College of Medicine/Montefiore Medical Center, Bronx, NY USA

## Introduction

HIV-1 cure remains elusive despite HAART due to the reservoirs of proviral DNA integrated into the human genome. Efforts to cure HIV-1 therefore need to aim at eliminating proviral DNA from cellular reservoirs. The first epigenetic signal identified in virus infected and uninfected cells has been promoter methylation. Compelling evidence confirms that specific promoter methylation can lead to gene silencing. Previous studies have examined HIV-1-epigenetics mostly in vitro.

## Materials and methods

We determined methylation patterns in HIV-1 proviral genomes from PBMCs obtained from 21 individuals with a spectrum of disease progression. The CpGs in the long terminal repeats (LTRs) of proviral DNA were investigated by bisulfite sequencing in up to 85 genomic variants per individual. This approach facilitates the study of the full range of CpG methylation and sequence variability of HIV-1 proviruses under conditions of natural selection in human populations.

## Results

In patients with advanced disease, the HIV-1 proviruses remained essentially unmethylated in their LTRs. In one long-term nonprogresssor, the percentage of methylated proviruses varied from 0-77% at different times after infection. More important and unexpected was the detection of three specific LTR-located CpG dinucleotides that had been selectively mutated to TpAs in >20 out of the 32 samples analyzed. Comparison to 11 HIV-1 LTR sequences in the Los Alamos HIV data base demonstrated that mutations in the sites identified by our study occurred more frequently than at other locations, although the mutations were different from TpAs.

## Conclusions

These specific CpGs, possibly including their abutting sequences, might indicate weak spots in the proviral genomes whose sacrifice by mutation to TpAs could enhance the HIV-1 potential for long-term proviral survival. These data suggest that the sites of the mutated CpGs occurring at conserved sites may serve as potential targets for therapeutic interventions to eliminate integrated proviruses.

(Grants: DFG-DO165/28-1; NIH-UO1-AI35004)

